# 36 A Regional Analysis of Completely Preventable Injuries Sustained from Burning Garbage and Yard Waste

**DOI:** 10.1093/jbcr/irad045.010

**Published:** 2023-05-15

**Authors:** Tina Boam, Abigail Rath, Sandra Fletchall, Sarah Sabbatini, Teresa Geib, Mamie Krebs, David Funk, Catherine Greer, Meghan Mincey, Mary Wayne, Sai Velamuri, Xiangxia Liu, David Hill

**Affiliations:** Firefighters Burn Center, Memphis, Tennessee; University of Tennessee Health Science Center, Memphis, Tennessee; Firefighters Burn Center, Memphis, Tennessee; Firefighters Burn Center, Memphis, Tennessee; Firefighters Burn Center, Memphis, Tennessee; Firefighters Burn Center, Memphis, Tennessee; Firefighters Burn Center, Memphis, Tennessee; Firefighters Burn Center, Memphis, Tennessee; Firefighters Burn Center, Memphis, Tennessee; Firefighters Burn Center, Memphis, Tennessee; University of Tennessee, Memphis, Tennessee; Firefighters Burn Center, Memphis, Tennessee; Regional One Health, Memphis, Tennessee; Firefighters Burn Center, Memphis, Tennessee; University of Tennessee Health Science Center, Memphis, Tennessee; Firefighters Burn Center, Memphis, Tennessee; Firefighters Burn Center, Memphis, Tennessee; Firefighters Burn Center, Memphis, Tennessee; Firefighters Burn Center, Memphis, Tennessee; Firefighters Burn Center, Memphis, Tennessee; Firefighters Burn Center, Memphis, Tennessee; Firefighters Burn Center, Memphis, Tennessee; Firefighters Burn Center, Memphis, Tennessee; University of Tennessee, Memphis, Tennessee; Firefighters Burn Center, Memphis, Tennessee; Regional One Health, Memphis, Tennessee; Firefighters Burn Center, Memphis, Tennessee; University of Tennessee Health Science Center, Memphis, Tennessee; Firefighters Burn Center, Memphis, Tennessee; Firefighters Burn Center, Memphis, Tennessee; Firefighters Burn Center, Memphis, Tennessee; Firefighters Burn Center, Memphis, Tennessee; Firefighters Burn Center, Memphis, Tennessee; Firefighters Burn Center, Memphis, Tennessee; Firefighters Burn Center, Memphis, Tennessee; Firefighters Burn Center, Memphis, Tennessee; University of Tennessee, Memphis, Tennessee; Firefighters Burn Center, Memphis, Tennessee; Regional One Health, Memphis, Tennessee; Firefighters Burn Center, Memphis, Tennessee; University of Tennessee Health Science Center, Memphis, Tennessee; Firefighters Burn Center, Memphis, Tennessee; Firefighters Burn Center, Memphis, Tennessee; Firefighters Burn Center, Memphis, Tennessee; Firefighters Burn Center, Memphis, Tennessee; Firefighters Burn Center, Memphis, Tennessee; Firefighters Burn Center, Memphis, Tennessee; Firefighters Burn Center, Memphis, Tennessee; Firefighters Burn Center, Memphis, Tennessee; University of Tennessee, Memphis, Tennessee; Firefighters Burn Center, Memphis, Tennessee; Regional One Health, Memphis, Tennessee; Firefighters Burn Center, Memphis, Tennessee; University of Tennessee Health Science Center, Memphis, Tennessee; Firefighters Burn Center, Memphis, Tennessee; Firefighters Burn Center, Memphis, Tennessee; Firefighters Burn Center, Memphis, Tennessee; Firefighters Burn Center, Memphis, Tennessee; Firefighters Burn Center, Memphis, Tennessee; Firefighters Burn Center, Memphis, Tennessee; Firefighters Burn Center, Memphis, Tennessee; Firefighters Burn Center, Memphis, Tennessee; University of Tennessee, Memphis, Tennessee; Firefighters Burn Center, Memphis, Tennessee; Regional One Health, Memphis, Tennessee; Firefighters Burn Center, Memphis, Tennessee; University of Tennessee Health Science Center, Memphis, Tennessee; Firefighters Burn Center, Memphis, Tennessee; Firefighters Burn Center, Memphis, Tennessee; Firefighters Burn Center, Memphis, Tennessee; Firefighters Burn Center, Memphis, Tennessee; Firefighters Burn Center, Memphis, Tennessee; Firefighters Burn Center, Memphis, Tennessee; Firefighters Burn Center, Memphis, Tennessee; Firefighters Burn Center, Memphis, Tennessee; University of Tennessee, Memphis, Tennessee; Firefighters Burn Center, Memphis, Tennessee; Regional One Health, Memphis, Tennessee; Firefighters Burn Center, Memphis, Tennessee; University of Tennessee Health Science Center, Memphis, Tennessee; Firefighters Burn Center, Memphis, Tennessee; Firefighters Burn Center, Memphis, Tennessee; Firefighters Burn Center, Memphis, Tennessee; Firefighters Burn Center, Memphis, Tennessee; Firefighters Burn Center, Memphis, Tennessee; Firefighters Burn Center, Memphis, Tennessee; Firefighters Burn Center, Memphis, Tennessee; Firefighters Burn Center, Memphis, Tennessee; University of Tennessee, Memphis, Tennessee; Firefighters Burn Center, Memphis, Tennessee; Regional One Health, Memphis, Tennessee; Firefighters Burn Center, Memphis, Tennessee; University of Tennessee Health Science Center, Memphis, Tennessee; Firefighters Burn Center, Memphis, Tennessee; Firefighters Burn Center, Memphis, Tennessee; Firefighters Burn Center, Memphis, Tennessee; Firefighters Burn Center, Memphis, Tennessee; Firefighters Burn Center, Memphis, Tennessee; Firefighters Burn Center, Memphis, Tennessee; Firefighters Burn Center, Memphis, Tennessee; Firefighters Burn Center, Memphis, Tennessee; University of Tennessee, Memphis, Tennessee; Firefighters Burn Center, Memphis, Tennessee; Regional One Health, Memphis, Tennessee; Firefighters Burn Center, Memphis, Tennessee; University of Tennessee Health Science Center, Memphis, Tennessee; Firefighters Burn Center, Memphis, Tennessee; Firefighters Burn Center, Memphis, Tennessee; Firefighters Burn Center, Memphis, Tennessee; Firefighters Burn Center, Memphis, Tennessee; Firefighters Burn Center, Memphis, Tennessee; Firefighters Burn Center, Memphis, Tennessee; Firefighters Burn Center, Memphis, Tennessee; Firefighters Burn Center, Memphis, Tennessee; University of Tennessee, Memphis, Tennessee; Firefighters Burn Center, Memphis, Tennessee; Regional One Health, Memphis, Tennessee; Firefighters Burn Center, Memphis, Tennessee; University of Tennessee Health Science Center, Memphis, Tennessee; Firefighters Burn Center, Memphis, Tennessee; Firefighters Burn Center, Memphis, Tennessee; Firefighters Burn Center, Memphis, Tennessee; Firefighters Burn Center, Memphis, Tennessee; Firefighters Burn Center, Memphis, Tennessee; Firefighters Burn Center, Memphis, Tennessee; Firefighters Burn Center, Memphis, Tennessee; Firefighters Burn Center, Memphis, Tennessee; University of Tennessee, Memphis, Tennessee; Firefighters Burn Center, Memphis, Tennessee; Regional One Health, Memphis, Tennessee; Firefighters Burn Center, Memphis, Tennessee; University of Tennessee Health Science Center, Memphis, Tennessee; Firefighters Burn Center, Memphis, Tennessee; Firefighters Burn Center, Memphis, Tennessee; Firefighters Burn Center, Memphis, Tennessee; Firefighters Burn Center, Memphis, Tennessee; Firefighters Burn Center, Memphis, Tennessee; Firefighters Burn Center, Memphis, Tennessee; Firefighters Burn Center, Memphis, Tennessee; Firefighters Burn Center, Memphis, Tennessee; University of Tennessee, Memphis, Tennessee; Firefighters Burn Center, Memphis, Tennessee; Regional One Health, Memphis, Tennessee; Firefighters Burn Center, Memphis, Tennessee; University of Tennessee Health Science Center, Memphis, Tennessee; Firefighters Burn Center, Memphis, Tennessee; Firefighters Burn Center, Memphis, Tennessee; Firefighters Burn Center, Memphis, Tennessee; Firefighters Burn Center, Memphis, Tennessee; Firefighters Burn Center, Memphis, Tennessee; Firefighters Burn Center, Memphis, Tennessee; Firefighters Burn Center, Memphis, Tennessee; Firefighters Burn Center, Memphis, Tennessee; University of Tennessee, Memphis, Tennessee; Firefighters Burn Center, Memphis, Tennessee; Regional One Health, Memphis, Tennessee; Firefighters Burn Center, Memphis, Tennessee; University of Tennessee Health Science Center, Memphis, Tennessee; Firefighters Burn Center, Memphis, Tennessee; Firefighters Burn Center, Memphis, Tennessee; Firefighters Burn Center, Memphis, Tennessee; Firefighters Burn Center, Memphis, Tennessee; Firefighters Burn Center, Memphis, Tennessee; Firefighters Burn Center, Memphis, Tennessee; Firefighters Burn Center, Memphis, Tennessee; Firefighters Burn Center, Memphis, Tennessee; University of Tennessee, Memphis, Tennessee; Firefighters Burn Center, Memphis, Tennessee; Regional One Health, Memphis, Tennessee

## Abstract

**Introduction:**

Burn injuries due to garbage and yard waste burning account for a high percentage of flame injuries in the south. Understanding the injury pattern and healthcare burden is important for effective prevention of these completely unnecessary burn injuries.

**Methods:**

This five-year retrospective, single-center, review included patients admitted between 2016 and 2021. Patients included were at least 14 years of age and sustained an open flame burn injury due to burning brush and/or trash. Patients who experienced a burn injury due to gasoline, hazardous material, lighter, fireworks, accelerant/gel, aerosol can, and burning bedding/clothing, were excluded.

**Results:**

Based primary residence of the 136 patients, 56% had access to free municipal waste disposal, 25% could have had access with additional payment (not included in taxes), and 18% did not have access to municipal waste disposal. The median age was 50 (32, 66.5) years. The majority were burning yard debris, while 32% were burning trash. The majority were male (85%) and Caucasian (71%). The median TBSA was 5 (2.5, 12) percent with 36% having some portion of full-thickness injury. One-third had some form of substance use with 29% tobacco, 5% alcohol, and 4% illicit drugs. There were 151 total operations with a median of 1 (0, 1.5) per patient. There were 1,620 unnecessary hospital days utilized (~6.6% of available bed-days per study period). The median length of stay was 3 (1, 10) days with the majority (85%) being discharged back to their home. Twenty-five percent were discharged with a paired functional status worse than pre-injury. Eighty-eight percent required no assistance prior to injury, while only 67% were discharged without requiring mobility or assistance with ADLs. Patients with some degree of pre-injury function limitations had a three-fold higher LOS (10 vs 3 days; p = 0.023) and associated costs. Although deaths were few in this sample, patients with lower pre-injury functionality had almost 5 times higher mortality (23.1% vs 5.3%; p = 0.083). Therapy data were available for 88 patients totaling 656 occupational and 462 physical therapy sessions, including acute care, inpatient rehabilitation unit, and outpatient. Unfortunately, there were 8 (6%) unnecessary deaths from their injuries with an average 77.3 ± 10.4 years of age and 42.4 ± 21.7% TBSA with 35.1 ± 23.7% being full-thickness. Total hospital charges exceeded $32.6 million with a median $32,952.26 ($8,790.48, $103,113.95) per patient. Payer source at day of discharge was mixed (private 37%, self-pay 26%, Medicare 20%, and Medicaid 17%).

**Conclusions:**

Injuries from waste burning are completely unnecessary and subsequent mortality, morbidity, and health care burden avoidable. Focusing future outreach efforts on education and resource availability may prevent future debris and trash related injuries.

**Applicability of Research to Practice:**

This is the first study to utilize repository data to analyze waste burning injuries from a regional perspective.

